# Understanding Associative Learning Through Higher-Order Conditioning

**DOI:** 10.3389/fnbeh.2022.845616

**Published:** 2022-04-18

**Authors:** Dilara Gostolupce, Belinda P. P. Lay, Etienne J. P. Maes, Mihaela D. Iordanova

**Affiliations:** Center for Studies in Behavioral Neurobiology, Department of Psychology, Concordia University, Montreal, QC, Canada

**Keywords:** associative learning, second-order conditioning, sensory preconditioning, memory integration, extinction

## Abstract

Associative learning is often considered to require the physical presence of stimuli in the environment in order for them to be linked. This, however, is not a necessary condition for learning. Indeed, associative relationships can form between events that are never directly paired. That is, associative learning can occur by integrating information across different phases of training. Higher-order conditioning provides evidence for such learning through two deceptively similar designs – sensory preconditioning and second-order conditioning. In this review, we detail the procedures and factors that influence learning in these designs, describe the associative relationships that can be acquired, and argue for the importance of this knowledge in studying brain function.

## Introduction

Understanding how stimuli occur relative to other stimuli in our environment is fundamental to making accurate predictions about the future and adapting behaviour accordingly. One way for such learning to ensue is to present stimuli together in time. For example, the painful experience of having been bitten by a dog can result in the development of fear of dogs causing one to avoid places whether dogs can be encountered. In the laboratory, this learning (i.e., first-order conditioning; Pavlov, 1927) is modeled using Pavlovian conditioning, which consists of pairings between a neutral sensory cue (or stimulus) such as a tone with an event of biological significance. While this form of learning accurately captures the formation of many associative relationships, it misses many others. Indeed, one need not directly experience event relationships in order to infer the likelihood of their occurrence in novel situations. To return to the example of the dog bite, one will likely not only avoid dogs (the stimulus directly associated with the aversive event) but also places where dogs frequent (e.g., parks, trails, your next-door neighbour’s yard) even though the bite had not occurred there. Here, the knowledge of where dogs can be encountered is integrated with the knowledge that dogs can cause painful bites. In other words, information acquired across different episodes or time points can be linked, thus offering an opportunity to infer unique event relationships and make novel predictions about the environment. Such integration is an example of the dynamic nature of memories, how memories become linked and how flexible behaviour is orchestrated [Holland, 1990; Gewirtz and Davis, 2000; Blaisdell, 2009; Seitz et al., 2021; for more on discontinuous events see Wallenstein et al. (1998) and Cai et al. (2016)].

Integration of distinct associative memories is elegantly captured in higher-order conditioning (e.g., Pavlov, 1927; Brogden, 1939). This learning consists of two conditioning episodes–one that leads to associative links between two neutral stimuli (i.e., S2→S1 where S2 could be an auditory cue such as a tone and S1 could be a visual cue such as a light) and another that links one of these stimuli (S1) with a biologically significant outcome (an appetitive or aversive unconditioned stimulus [US], i.e., S1→US). Subsequent presentations of S2 reveal its ability to invigorate conditioned responses (CRs) indicative of expectation of the US. This form of learning is termed higher-order because S2 is never directly paired with the US. Rather, it engages conditioned responding by virtue of its pairing with S1 which was directly associated with the US. That is, S2 acquires value through an intermediary. This learning requires integration of the sensory learning phase with the fear conditioning phase and provides a mechanism through which value (be it aversive or appetitive) can propagate across the memory network through higher-order associative links.

## Procedures and Factors That Influence Higher-Order Conditioning

There are two classic designs of higher-order conditioning, namely, sensory preconditioning and second-order conditioning. While both types of designs consist of the same learning phases, that is, sensory training and appetitive or aversive conditioning (as outlined above), the order of the phases is reversed. In sensory preconditioning, S2→S1 pairings precede S1→US pairings, whereas in second-order conditioning S1→US pairings precede S2→S1 pairings. Although, the order of the learning phases may seem like a minor difference in experimental design, it is of tremendous importance because it governs what is learned during these distinct forms of higher-order conditioning (see below). Accounts of higher-order learning were originally reported by Pavlov (1927) where cues directly paired with an appetitive or aversive outcome could support the acquisition of secondary conditioned reflexes when paired with novel cues in the absence of the associated outcome (i.e., second-order conditioning). In both humans and animals, Prokofiev and Zeliony (1926) reported that sensory pairings between two cues followed by aversive conditioning of one of those cues led to fear of the other (indirectly paired) sensory cue (i.e., sensory preconditioning). This was subsequently investigated more thoroughly by Brogden (1939), coining the term “sensory preconditioning.” While these forms of higher-order learning have been replicated numerous times across species including drosophila, goldfish, pigeons, mice, rats, rabbits, monkeys, and humans (e.g., Reid, 1952; Rizley and Rescorla, 1972; Rashotte et al., 1977; Pfautz et al., 1978; Rescorla, 1979; Amiro and Bitterman, 1980; Cook and Mineka, 1987; Beauchamp and Gluck, 1988; Gibbs et al., 1991; Müller et al., 2000; Brembs and Heisenberg, 2001; Mead and Stephens, 2003; Tabone and de Belle, 2011; Lee and Livesey, 2012; Busquets-Garcia et al., 2017; Renaux et al., 2017; Craddock et al., 2018; Wong and Pittig, 2022), the precise design parameters employed can easily influence the strength and content of learning. Below, we enumerate a list of design factors that have been reported in the literature along with their associated influence on higher-order conditioning. This information has also been summarized in Table 1.

**TABLE 1:** Procedures and factors that influence higher-order conditioning.

Factors	Examples	Influence on learning
Stimulus type	Auditory (e.g., tone, white noise, clicker)	Associability and similarity between S2 and S1 influence the strength of higher-order conditioning.
	Visual (e.g., flashing light, key light, context)	
	Odour (e.g., almond, vanilla)	
	Flavour (e.g., sucrose, saline)	
	Shape (e.g., rectangle, triangle)	
	Appetitive US (e.g., food pellets, sucrose pellets)	
	Aversive US (e.g., footshock, illness)	

Stimulus arrangement	Serial (i.e., S2 offset coincides with S1 onset) Simultaneous (i.e., S2 and S1 presented at the same time)	Simultaneous arrangement results in superior sensory preconditioning effect relative to serial arrangement (Thompson, 1972; Rescorla, 1980b; Holland and Ross, 1983). Both serial and simultaneous S2-S1 pairings produce robust second-order learning, however, the arrangement has a differential effect on the content of learning.

Stimulus similarity	S2 and S1 chosen from the same stimulus type S2 and S1 chosen from different stimulus type	Pairing of similar stimuli proceed more rapidly relative to dissimilar stimuli in second-order conditioning (Rescorla and Furrow, 1977; Rescorla and Cunningham, 1979). Spatial similarity and using same cue modality promote sensory preconditioning (Holland, 1977; Rescorla and Cunningham, 1979)

Stimulus order	Forward serial order (i.e., S2 precedes S1) Backward serial order (i.e., S1 precedes S2, US precedes S1)	Higher-order conditioning designs classically use forward serial pairings (Pavlov, 1927). However, backward serial pairings of S1 and S2; US and S1 also support learning (Barnet et al., 1997; Ward-Robinson and Hall, 1998)

Trial number	Conditioned aversion: Single S2-S1 trial Aversive: 4 serial S2-S1 trials, 8 serial S2-S1 trials (Parkes and Westbrook, 2010) Appetitive: 100 trials (Rashotte et al., 1977) 40 trials (Holland and Rescorla, 1975a) 200 trials (Reid, 1952) 2 trials (Jones et al., 2012; Sadacca et al., 2018)	Sensory preconditioning and second-order conditioning can be obtained in single S2 and S1 pairing in conditioned aversion preparation (Archer and Sjöden, 1982). Aversive higher-order learning proceeds in four trials for second-order conditioning and eight trials for sensory preconditioning (Parkes and Westbrook, 2010). In contrast, appetitive designs may require more training trials (Reid, 1952; Rashotte et al., 1977; Jones et al., 2012; Sadacca et al., 2018).

Reinforced presentations	S2-S1 pairing followed by US delivery	Second-order learning can be obtained by reinforced S2→S1 pairings following S1 training (Leidl et al., 2018; Williams-Spooner et al., 2019).

### Stimulus Type

Various stimuli have been used in higher-order conditioning experiments including colour (Rashotte et al., 1977), shape (Rescorla, 1980a), odour (Holland, 1981), flavour (Holland, 1981, 1983), auditory cues such as tone (Rizley and Rescorla, 1972), white noise (Holland and Ross, 1983), clicker (Ward-Robinson and Hall, 1998) and visual cues such as key light (Rashotte et al., 1977), flashing light (Parkes and Westbrook, 2010; Wong et al., 2019), and context (Archer and Sjöden, 1982; Helmstetter and Fanselow, 1989; Iordanova et al., 2008). The types of USs used in higher-order designs are similar to those used in first-order conditioning studies including footshocks, rewards such as food to a hungry rat, lithium chloride (LiCl)—induced illness (e.g., Rizley and Rescorla, 1972; Holland and Rescorla, 1975a; Archer and Sjöden, 1982; Ward-Robinson and Hall, 1998). Other aspects of stimulus type such as the intensity of the US with which S1 is paired, and the physical similarity between S2 and S1 (Garcia and Koelling, 1966; Rescorla and Furrow, 1977; Rescorla, 1980a) influence the strength of higher-order conditioning.

### Stimulus Similarity

An important contributing factor to learning in higher-order conditioning is stimulus similarity. Specifically, when similar stimuli are used in the roles of S2 and S1, higher-order conditioning is facilitated compared to using dissimilar stimuli. Rescorla and Furrow (1977) showed that second-order conditioning proceeded more rapidly when S1 and S2 belonged to the same, compared to different, class of stimuli (e.g., colour: blue or green; orientation: horizontal or vertical lines). These effects were not due to stimulus generalization or pseudo-conditioning (Rescorla and Furrow, 1977). Cue similarity also facilitates second-order conditioning when the cues form a part-whole relationship. For example, in a pigeon autoshaping design, Rescorla (1980a) used achromatic shapes (triangle or square) as S2 and red shapes (triangle or square) as S1. Congruency in the shape, that is, when the achromatic shape was the same as the coloured shape, resulted in better second-order conditioning. Similar effects were reported in sensory preconditioning (Holland and Ross, 1983) and in appetitive second-order conditioning (Holland, 1977) using same cue modality or spatial similarity (Rescorla and Cunningham, 1979).

### Stimulus Arrangement

The sensory cues used in higher order conditioning designs can be presented simultaneously or serially. Simultaneous presentations of S1 and S2 refer to instances when the cues are presented in compound such that they overlap. In serial presentations, S1 tends to follow S2 such that S2 offset often coincides with S1 onset. Although learning accrues to S2 in both scenarios, the temporal arrangement influences the association acquired by the higher-order S2. In sensory preconditioning, simultaneous presentation of stimuli during sensory training results in superior learning compared to serial S2→S1 pairings (Thompson, 1972; Rescorla, 1980b; Holland and Ross, 1983). This effect can be explained when considering the associations that form between the cues during sensory training. Simultaneous presentations facilitate associations between the sensory characteristics of S2 and S1 rather than a predictive relationship between them (i.e., S2 predicts S1 presentation). The latter is favored by a serial arrangement. Second-order conditioning is also achieved using both simultaneous and serial S2 and S1 presentations. Rescorla (1982) showed that both serial and simultaneous arrangements result in similar levels of second-order conditioning, but the arrangement has a differential effect on what is learned (see below).

### Stimulus Order

In studies where the cues have been presented serially in sensory preconditioning or second-order conditioning, it is common for S2 to precede S1. However, instances of S1 preceding S2 (i.e., S1→S2) are also effective in supporting learning. In an aversive design, sensory preconditioning was successfully obtained using such a serial *backward* order (i.e., S1→S2; Ward-Robinson and Hall, 1998). Reversing the order during first-order conditioning (i.e., the US preceded S1) also resulted in robust sensory preconditioning and second-order conditioning, in a lick suppression preparation with rats (Barnet et al., 1997).

### Trial Number

The number of trials used to establish higher-order conditioning depends on various factors including the nature of the design (e.g., fear, reward, taste aversion), cue modality, stimulus arrangement, the model organism (e.g., rat, pigeon, rabbit), and the response measure (e.g., magazine approach, freezing, conditioned suppression). Higher-order fear conditioning progresses fairly rapidly: four trials of serial S2→S1 pairings is sufficient to obtain second-order learning (Rizley and Rescorla, 1972; Parkes and Westbrook, 2010; Lay et al., 2018) and sensory preconditioning can be achieved in eight serial S2→S1 trials (Rizley and Rescorla, 1972; Parkes and Westbrook, 2011; Wong et al., 2019). Higher-order conditioning designs involving rewards require more extensive S2→S1 training. In particular, second-order conditioning is successful using 100 trials across 10 days in pigeons (Rashotte et al., 1977), or 40 trials across four days in rats (Holland and Rescorla, 1975a) whereas sensory preconditioning has been obtained with 200 trials across 10 days in pigeons (Reid, 1952), but with as few as 12 trials across two days in rats (Jones et al., 2012; Sadacca et al., 2018).

The large number of trials often required for second-order conditioning can have unintended effects. As the number of S2→S1 trials increase in second-order conditioning, responding to S2 decreases, which is in contrast with the increase in responding to S1 across S1→US pairings. When S2→S1 pairings are alternated with continued S1→US pairings, the S2 can become a signal for the absence of the US (Herendeen and Anderson, 1968; Rescorla et al., 1973; Holland and Rescorla, 1975b; Yin et al., 1994). That is, conditioned inhibition to S2 accrues, competing with its ability to exhibit second-order conditioning (Gewirtz and Davis, 2000; Parkes and Westbrook, 2010). In a lick suppression study in rats, 20 simultaneous S2→S1 pairings favored conditioned inhibition over second-order conditioning and a hundred such trials rendered S2 a conditioned inhibitor regardless of whether S2 and S1 were paired simultaneously or serially (Stout et al., 2004). The transition of S2 from a second-order excitor to a conditioned inhibitor was quicker when S2 and S1 were presented in compound (Stout et al., 2004). To limit the development of conditioned inhibition in second-order conditioning, fewer S2→S1 pairing should be employed. This is possible in conditioned taste aversion. Indeed, a single pairing between a gustatory S2 and a contextual S1 was sufficient to obtained sensory preconditioning and second-order conditioning provided the US used to conditioned S1 was very salient (i.e., LiCl; Archer and Sjöden, 1982). These data, among others, reveal the importance of the strength of S1→US association on higher-order conditioning (Bond and Harland, 1975; Bond and Di Giusto, 1976).

### Reinforced Presentations

Some instances of second-order fear conditioning consist of reinforced serial S2→S1 pairings following S1 training [i.e., S2→S1→US; Williams-Spooner et al., 2019; see also Mahmud et al. (2019)]. This design, like the standard non-reinforced design, results in robust learning about the second-order stimulus relative to an unpaired control (Leidl et al., 2018; Williams-Spooner et al., 2019). In reward learning, reinforced serial S2→S1 presentations lead to higher level of responding during training compared to non-reinforced S2→S1 presentations (Holland, 1980). This effect, however, was likely due to the development of S2→US associations (Holland, 1980). To show this, Holland (1980) tested S2 under conditions that reveal the strength of second-order associations (i.e., under food satiation) and reported lower level of responding to S2 when trained in the reinforced serial case. Holland (1980) further showed that surprising food presentations or omissions were more detrimental to second-order conditioning than when such events were expected. This was taken as evidence for the role of outcome interference in the development S2→S1 associations, which was successfully alleviated by delaying outcome delivery (Holland, 1980).

## Response Measures in Higher-Order Conditioning

In first-order conditioning, an aversive US (e.g., a mild electric shock) conditions species-specific defensive behaviours (e.g., freezing, Blanchard and Blanchard, 1969; Bolles, 1970; Fanselow, 1980) or conditioned suppression (e.g., Rescorla and Furrow, 1977; Bouton and Bolles, 1980), whereas an appetitive US (e.g., sucrose pellets) supports conditioned approach (e.g., Holland, 1977). The US, however, is not the only determinant of conditioned responses. Auditory and visual cues can support cue-based responses including rearing, head jerk, perambulation, and general activity (Holland and Rescorla, 1975a,b; Holland, 1977, 1984). While auditory stimuli elicit startle and head jerk, visual stimuli elicit rearing (Holland, 1977). Startle and rearing are considered orienting responses (OR) and are seen to novel but not familiar non-reinforced cues and maintained or augmented to cues that have undergone conditioning. Head jerk is specific to conditioned auditory cues. ORs and CRs are differentially distributed across the duration of a conditioned stimulus, with ORs occurring mostly during the beginning of visual cues and food-cup CRs following afterwards, while CRs and ORs elicited by auditory cues are more evenly distributed (Holland, 1977; Hatfield et al., 1996). In second-order conditioning, pairing an auditory S2 with either a visual or auditory S1 leads to similar proportions of CRs and ORs, with head jerk being the predominant response to S2 in both cases (Holland, 1977).

## Content of Higher-Order Conditioning

The associative links that govern sensory preconditioning and second-order conditioning differ depending on the procedural details. As different designs are often used to study the neural substrates of higher-order learning, it is imperative that one is aware that procedural differences can lead to differences in associative content (i.e., what is learned; see also Gewirtz and Davis, 2000; Parkes and Westbrook, 2011; Gostolupce et al., 2021). We cover these below.

### Extinction of S1

The first evidence to highlight the differences in learning between sensory preconditioning and second-order conditioning came from Rizley and Rescorla (1972). In a fear conditioning procedure with footshock as the US, the authors showed that reduction in responding to S1 *via* repeated presentations of this cue in the absence of the US (i.e., S1 extinction training) consequently reduced responding to S2 in sensory preconditioning but not in second-order conditioning [see also Parkes and Westbrook (2010), Holmes et al. (2014)]. Similar findings have been reported in higher-order reward conditioning by Holland and Rescorla (1975a) as well as in a conditioned taste aversion design (Archer and Sjöden, 1982). These data provide convincing evidence that the association between S2 and S1 is key to regulating sensory preconditioning but not second-order conditioning (Rizley and Rescorla, 1972; Holland and Rescorla, 1975a).

It turns out, however, that the arrangement of stimulus presentation or stimulus similarity can influence the nature of associative learning in second-order conditioning. Rescorla (1982) showed that simultaneous S2→S1 pairings produce second-order responding that is sensitive to S1-extinction. This may be because simultaneous presentations lead to within compound associations or the development of an S2S1 configural unit (Pearce, 1994, 2002), meaning that the sensory cues can activate representations that contain one another.

It is also possible to obtain second-order responding that is sensitive to extinction of S1 when S2 and S1 belong to the same cue modality (i.e., both S2 and S1 as auditory cues such as tones of different frequencies or as visual cues such as a flashing houselight or a jeweled signal light; Rescorla and Furrow, 1977). In an autoshaping procedure, pigeons were first trained to peck a white key light S1 by pairing it with grain delivery (Rashotte et al., 1977). A blue key light S2 was trained with S1 in a serial manner to achieve second-order conditioning to S2. Extinction of S1 resulted in disruption of second-order key peck responding. These findings demonstrate that second-order conditioning is sensitive to manipulations of S1 when S2 and S1 belong to the same cue modality (but see Experiment 4 using different modalities in which S1 is an operant discriminant).

Determining what is learned during second-order conditioning is further informed by the behaviours that are measured. While extinction of a visual S1 after second-order conditioning leaves intact food-cup approach and head jerk CRs to an auditory S2 (Holland and Rescorla, 1975a), rear ORs to S2 are abolished (Setlow et al., 2002; McDannald et al., 2013). Given that rearing is generally only evoked by either visual cues or S2s by virtue of them being paired with a visual S1, this OR is thought to represent the behavioural readout of a S2→S1 (i.e., stimulus→stimulus) association (Setlow et al., 2002). This suggests that an S2→S1 association may be formed, but such associations are unlikely to drive the conditioned responses normally measured in second-order conditioning.

Finally, in a series of clever studies that used the nature of responding to determine the nature of the associations between events in second-order conditioning, Holland (1977) revealed that S2 is likely linked to the affective or motivational state induced by the US. Specifically, he examined whether an auditory S2 would acquire auditory ORs or visual ORs when paired with either an auditory or visual S1 in a second-order design. The data confirmed that an auditory S2 elicits an auditory-specific response and does not become associated with the cue-based response elicited by S1, eliminating the likelihood of a S2→CR (i.e., stimulus→response) association. Holland’s interpretation was further confirmed by Winterbauer and Balleine (2005) who showed that second-order cues enter into associations with the specific motivational aspects of the US in water- and food-deprived rats and that this learning was not dependent on the motivational state at the time of training.

### Devaluing the US

Although S2 is never directly paired with the US in higher-order designs, some evidence suggests that a S2→US association must not be discounted. Indeed, the development of associations between actual and associatively evoked stimuli are well-supported by the literature (e.g., Holland, 1981, 1983; Holland and Forbes, 1982; Iordanova et al., 2008; Lin and Honey, 2010, 2011, 2016; Wong et al., 2019). To account for such learning, Holland (1981, 1983) proposed a modification to Wagner’s Standard Operating Procedures (SOP; Wagner, 1981). Briefly, according to SOP, stimuli can be in three states of activation, a focal A1 state, a working memory A2 state and an inactive I state. Excitatory conditioning occurs when events are concurrently in an A1 state of activity whereas inhibitory conditioning occurs when a cue is in an A1 state and the US in an A2 state. In a series of conditioned taste aversion experiments, Holland (1981, 1983) provided evidence that excitatory learning also occurs between two events (e.g., food and LiCl) when the food is in A2 (i.e., associatively activated) and the LiCl in A1 (physically present). This proposal accounts for the development of S2→US associations in sensory preconditioning designs because S1 would place its associate S2 into an A2 state during S1→US training while the US is in an A1 state, thereby allowing for S2→US learning in this phase.

To determine whether the representation of the US is linked to S2, Holland and Rescorla (1975a) used a devaluation procedure. They showed that reducing the value of an appetitive US led to a corresponding reduction in responding to S2 in sensory preconditioning but not second-order conditioning. This was also confirmed by Rescorla (1973) who devalued an aversive US (loud noise) using habituation. In other words, S2 is not linked to the US in second-order conditioning, at least early on in S2→US training. The lack of devaluation effects in second-order conditioning is consistent with the original stipulation of SOP (Wagner, 1981), which holds that during S2→S1 pairings, S2 would be in an A1 state whereas the US would be associatively evoked by S1 and therefore in an A2 state, resulting in the development of S2 as a conditioned inhibitor for the US. As mentioned, conditioned inhibition can accrue to a second-order S2 when the number of S2-S1 pairings increases and these trials are alternated with continued S1-US pairings (Herendeen and Anderson, 1968; Rescorla et al., 1973; Holland and Rescorla, 1975b; Yin et al., 1994), lending support for the SOP proposal. Intriguingly, this inhibitory association can co-exist with the excitatory second-order association (Holland and Rescorla, 1975b) and is greater when stimuli are similar compared to dissimilar (Rescorla, 1980a).

In summary, the studies reviewed above show that higher-order conditioning can be obtained using a variety of stimuli under diverse conditions. While sensory preconditioning is supported by associations between S2 and S1 as well as between S2 and the associatively evoked US, those that drive second-order conditioning are parameter-dependent. In the somewhat classic serial design that uses cues of different modalities, second-order conditioning is dependent on S2→motivational state associations and not on S2→S1 (evidenced using S1 extinction), nor S2→US (evidenced using US devaluation), nor S2→CR (evidenced in the inability of S2 to acquire cue-specific responses indicative of S1 expectation) associations. Altering the cue arrangement or modality, shifts the content of what is learned.

## Implications for Neuroscience

The quest for uncovering the neural mechanisms of higher-order learning is gaining momentum [e.g., Iordanova et al., 2009, 2011a,b; Horne et al., 2010; Jones et al., 2012; Wimmer and Shohamy, 2012; Holmes et al., 2013; Holland and Hsu, 2014; Holland, 2016; Lin and Honey, 2016; Sadacca et al., 2018; Wong et al., 2019; Hart et al., 2020; for reviews see Parkes and Westbrook (2011), Fournier et al. (2021), Gostolupce et al. (2021), and Holmes et al. (2021)]. Indeed, dissociations in the neural mechanisms of sensory preconditioning and second-order conditioning have been reported (Parkes and Westbrook, 2010; Holmes et al., 2013; Holland and Hsu, 2014; Holland, 2016) as have been nuances in the regulation of cue- vs. outcome-based responses elicited by higher-order stimuli (Gallagher et al., 1990; Hatfield et al., 1996; McDannald et al., 2013). These lines of evidence suggest that different neural areas regulate different types of associations despite the similarity in training, and that parallel systems drive subsets of behavioural responses established under the same training conditioning.

Our understanding of the functional role of distinct neural substrates can be greatly advanced by the study of higher-order learning. These preparations expand the conditions under which learning occurs, extending our study of how the brain learns. In addition, they provide important information into the associative structures that control behaviour, thereby offering particular insight into the function of brain areas that regulate this learning. This manuscript reviews the distinct procedures and parameters that supports higher-order learning and how this affects the corresponding associative architecture, which we hope will bolster the field’s analysis of the corresponding neural architecture.

## Author Contributions

All authors contributed to the article by conceiving the idea for the manuscript and developing the narrative. All authors approved the submitted version.

## Conflict of Interest

The authors declare that the research was conducted in the absence of any commercial or financial relationships that could be construed as a potential conflict of interest.

## Publisher’s Note

All claims expressed in this article are solely those of the authors and do not necessarily represent those of their affiliated organizations, or those of the publisher, the editors and the reviewers. Any product that may be evaluated in this article, or claim that may be made by its manufacturer, is not guaranteed or endorsed by the publisher.
